# The first direct replication on using verbal credibility assessment for the detection of deceptive intentions

**DOI:** 10.1002/acp.3439

**Published:** 2018-07-16

**Authors:** Bennett Kleinberg, Lara Warmelink, Arnoud Arntz, Bruno Verschuere

**Affiliations:** ^1^ Department of Psychology University of Amsterdam Amsterdam The Netherlands; ^2^ Department of Psychology Lancaster University Lancaster UK

**Keywords:** deception detection, intentions, replication, verbal credibility assessment

## Abstract

Verbal deception detection has gained momentum as a technique to tell truth‐tellers from liars. At the same time, researchers' degrees of freedom make it hard to assess the robustness of effects. Replication research can help evaluate how reproducible an effect is. We present the first replication in verbal deception research whereby ferry passengers were instructed to tell the truth or lie about their travel plans. The original study found truth‐tellers to include more specific time references in their answers. The replication study that closely mimicked the setting, procedure, materials, coding, and analyses found no lie–truth difference for specific time references. Although the power of our replication study was suboptimal (0.77), Bayesian statistics showed evidence in favor of the null hypothesis. Given the great applied consequences of verbal credibility tests, we hope this first replication attempt ignites much needed preregistered, high‐powered, multilab replication efforts.

## 1 INTRODUCTION

In the challenge to tell truth‐tellers from liars, verbal deception detection has emerged as one of the more promising approaches (Oberlader et al., 2016; Vrij, Fisher, & Blank, 2015). Verbal deception detection sets out to identify verbal indicators of deception in statements made about an event. Based on the notion that liars will have more difficulty providing a convincing and hence detailed account of a fabricated event than truth‐tellers, the cognitive approach to deception postulates that the differences in difficulty are represented in, for example, the richness of the verbal account about the event (Vrij et al., 2015). Similarly, the theory of Reality Monitoring poses that the content of a statement about a genuinely experienced event can be recalled in more detail than the content of a fabricated event (Johnson, Bush, & Mitchell, 1998). Both the cognitive approach and Reality Monitoring agree on the prediction that truthful statements are richer in detail than deceptive statements. There is a body of research on the verbal deception detection approach with meta‐analytical findings suggesting that detail richness can identify liars and truth‐tellers better than chance (Masip, Sporer, Garrido, & Herrero, 2005; Oberlader et al., 2016; Vrij et al., 2015). However, meta‐analyses rely on the quality of the original studies and cannot ascertain whether the individual effects reported in studies are reliable (van Elk et al., 2015). For progress in the field of verbal deception detection, replication studies are needed to solidify the findings and to work towards a strong empirical fundament that practitioners can apply. In other words, replication efforts are just as important as new, exploratory studies: “innovation points out paths that are possible; replication points out paths that are likely; progress relies on both” (Open Science Collaboration, 2015, p. 7).

### 1.1 Replicating verbal deception detection research

The importance of replication research was shown by a landmark finding that only one third to one half of 100 psychological experiments replicated (Open Science Collaboration, 2015). A replication study can be conceptual or direct (Nosek et al., 2015). Conceptual replication studies are those that test a previously found effect under new circumstances, taking into account the key ingredients that are believed to matter. A direct replication aims to mimic the original study as closely as possible (Simons, 2014). For any effect to matter, it should be obtainable under similar circumstances. That is if experiment *X* finds an effect, a new experiment *Y* following the procedure, sample size, and analysis of *X* should be able to see that same effect. The field of verbal deception detection is characterized by a multitude of interviewing techniques (e.g., asking difficult questions vs. open recall), cues (e.g., plausibility, consistency, and richness of detail), coding of those cues (e.g., what counts as a detail), annotation methods (e.g., manual human annotation and automated information extraction), and analytical approaches (e.g., individual cues vs. predictive modelling with multiple cues). These elements allow for high researchers' degrees of freedom (Gelman & Loken, 2013), that is, aspects on which the researcher has to make decisions when conducting a study and presenting results. The resulting variation between studies makes it hard to assess how robust the effects found in verbal deception detection are. In the current paper, we, therefore, present the first replication of verbal deception detection research.

### 1.2 The original study

We aimed to replicate the second experiment of Warmelink, Vrij, Mann, and Granhag (2013). Eighty‐four participants (36 male; mean age 58 years, *SD* = 12.6) were instructed to either tell the truth or lie about the reasons for travelling on a 6‐hr‐long ferry trip between Portsmouth (UK) and Caen (France). Participants were approached by an interviewer blind to the experimental condition and were asked either a control question (“Please describe in as much detail as possible what you are going to do today at your destination”) or a temporal prompt question (“Please describe what your timetable is for today at your destination”). The answers (word count *M* = 33.7, *SD* = 20.71) were manually annotated by two independent, trained human judges on specific times (e.g., “half past seven” and “five o'clock”), temporal details (e.g., “earlier” and “1 hour”), and spatial details (e.g., “in Paris” to “to London”). Truthful answers contained more mentions of specific times than deceptive answers (*d* = 0.54; 95% confidence interval [CI]: 0.10; 0.99). We chose the effect found for specific times for replication because (a) the very short interview (47 s) is attractive for applied purposes, (b) the dependent measure of specific times is well‐automatable (Kleinberg, Mozes, Arntz, & Verschuere, 2017), and (c) the effect size is promising for a field characterized by relatively small effects (DePaulo et al., 2003).

### 1.3 The current study: Direct replication part

We replicated the time prompt question findings from the second experiment in Warmelink et al. (2013). The study was conducted on a ferry on the Dutch islands, and participants were interviewed in Dutch. We extended the original experiment to further test whether actively eliciting specific information benefitted deception detection. Note that the additional question came after the replication part so that it could not affect the replication. Our first hypothesis is directly taken from the original study and states that truthful answers to the time schedule question contain a higher proportion of specific time occurrences than deceptive answers.

### 1.4 The current study: Additional question and coding

Apart from the direct replication part, we also examined whether the proportion of spatial details is higher in truthful than deceptive answers on an additional route description question. Similar to the prompt question mechanism for specific time references in the original study (i.e., asking for specific times enlarges truth–lie differences), asking for a route description might be helpful to invoke truth–lie differences on an additional dimension, namely, spatial details. Because the majority of verbal deception research resorts to humans who count the occurrences of verbal indicators, we further added two conceptually identical hypotheses on the related, computationally extracted constructs (temporal and spatial details). We expected that the proportion of “time” and “space” references as extracted with word count software is higher in truthful than in deceptive answers for questions on the respective domain (i.e., the time schedule and the route question). The procedure, manipulations, hypotheses, and analyses for the current study were preregistered before data collection (accessible at https://osf.io/w9qe2/register/565fb3678c5e4a66b5582f67). The materials, data, and code are available at https://osf.io/t29dz/. This paper reports all measures, conditions, data exclusions, and considerations to determine the sample size as stated in the preregistration.

## 2 METHOD

### 2.1 Participants

We approached participants on a ferry and interviewed them about their plans at their destination. We collected data from passengers on the ferry from the Dutch mainland (Harlingen) to the Dutch island Terschelling, which took approximately 120 min. Similar to the original study, willingness to participate was high with more than 80% of approached participants agreeing to partake. We aimed to collect data for the identical sample size as the original study (*n* = 84). As stated in the preregistration, this sample size is nearly identical to the one reached with a priori statistical power analysis for the key to‐be‐replicated effect size of *d* = 0.54 (one‐sided *t* test, alpha significance level 0.05, and power of 0.80, required *n* = 88). Our initial sample consisted of 85 participants, of whom six were excluded because they did not follow the instructions properly (e.g., they were not lying in the deceptive condition). Our final sample consisted of 79 participants, randomly assigned to either the truthful (*n* = 41, 39.47% female, *M*_*age*_ *=* 45.51 years, *SD*_*age*_ = 18.39) or deceptive condition (*n* = 38, 41.46% female, *M*_*age*_ *=* 45.95 years, *SD*_*age*_ = 14.68). There was no difference between the two conditions in gender, *X* ^2^(1) = 1.00, *p* = 0.999, or age, *F*(1, 77) = 0.01, *p* = 0.908.

### 2.2 Design

The design of this experiment is 2 (Veracity: truthful vs. deceptive, between‐subjects) by 2 (Question focus: time schedule vs. route description, within‐subjects) with the proportion of human‐coded specific times as key dependent variable. The focus of the replication is on the time schedule questions identical to the original study's “time prompt” condition.

Additional dependent variables—as outlined in the preregistration—are the proportion of human‐coded spatial details, as well as the automatically coded proportion of temporal and spatial details. As a control, we also asked for participants' motivation to be convincing.

### 2.3 Procedure

Two experimenters gathered the data on the ferry boat on 4 days in 2017. The experimenters approached participants for voluntary participation in a “deception detection experiment.” All experimenter–participant interaction was in Dutch. Experimenter 1 approached the participants, asked whether they were willing to participate, and had them sign the informed consent form. Before participants were allocated to either the truthful or deceptive condition, Experimenter 1 established the ground truth by asking the participants what their plans at their destinations were (e.g., “going home” and “weekend trip to Terschelling”) and asked for the participants' age and whether they had made the trip before. All participants were randomly assigned to the truthful or deceptive condition—participants chose an envelope from a shuffled stack of all envelopes containing the instructions for truth‐tellers or liars. The participants read the instructions according to their condition in the envelope as follows: “*You are in the truth condition. In a few minutes, an interviewer will ask you a few questions about your trip. Your task is to tell the truth about what you are going to do at your trip's destination. Try to convince the interviewer that you are telling the truth. There will be no follow‐up questions*” (truthful condition); and “*You are in the lie condition. In a few minutes, an interviewer will ask you a few questions about your trip. Your task is to lie about what you are really going to do at your trip's destination and to pretend that you are travelling for a different reason. Try to convince the interviewer that you are telling the truth. There will be no follow‐up questions*” (deceptive condition).

Each participant had 3 min of preparation time before the second experimenter (i.e., the interviewer) arrived.

The interview consisted of two brief questions. The first one (time schedule question) was focused on the temporal aspects of the journey, and the interview question was identical (translated to Dutch) to the one asked in the original experiment: “Please describe in as much detail as possible what your timetable is for today at your destination.”

The additional question that we added (route description question) targeted spatial aspects of the trip and concerned the route description from the moment the participant got off the ferry boat to their destination (“Please describe the route from when you leave the boat to your destination”). Each interview was audio‐recorded and later transcribed. After the interview, the experimenter asked for the participants' motivation to provide a convincing story (from 1—very low—to 10—very high), to recall their veracity instructions, and noted the participants' gender.

### 2.4 Human coding of statements

The transcribed interviews were coded by two independent and trained human judges. Before coding the actual transcripts, both coders received a detailed 3‐hr training session on practicing statements from a different study (but also on truthful and deceptive intentions) with one of the authors (B. K.). The annotation guidelines were identical to those used in the original study. After discussing annotation inconsistencies, the two judges annotated another six full statements of which the annotation was approved by the lead author of the original study and coauthor of the current paper (L. W.). We instructed the coders to annotate and count the number of specific time occurrences (e.g., “quarter past one”) using verbatim the same instructions from the original experiment. For the additional hypothesis and the exploratory part, the coders also counted the number of spatial details (e.g., “next to” and “down”) and the number of temporal details (e.g., “after,” “before,” and “subsequently”). To assess the reliability of the coding procedure, we had the first coder score 40% of the statements and the second coder score all statements. The agreement between the two human judges was high (specific time: Pearson correlation coefficient *r* = 0.90, intraclass correlation *ICC* = 0.86, *p* < 0.001; spatial details: *r* = 0.92, *ICC* = 0.89, *p* < 0.001; temporal details: *r* = 0.68, *ICC* = 0.71, *p* < 0.001). For the analysis, we used the judgments of the second coder and standardized the count variables (specific times, spatial, and temporal details) by the word count of each statement per question type (see Table 1 for examples high and low in human coded variables).

**Table 1:** Examples of statements high and low in specific times and spatial details human coding

	High	Low
Specific times (Question 1)	“I arrive at circa **twelve o'clock**, then I'll unpack and make my room. […] Then, at **three o'clock**, I'll start working until **half past four**. Then we will all quickly have a small bite and work until **nine o'clock**.”	“Well, I'll arrive soon and will then rent a bike to at the tourist office. I'll cycle until **quarter to four** and then go back to the mainland. Then I will go home. […]”
Spatial details (Question 2)	“Ehm. I arrive **in** West‐Terschelling. There I'll step **off** the ferry and walk **along** the Hoofdweg [streetname] through the small villages **to** Midsland. […]”	“Driving the car as fast as we can. No, just joking, I don't want another fine. We take the car **to** Elst.”

*Note*. The respective category coding is highlighted in bold.

### 2.5 Automated coding of statements

An alternative to human judgments is the Linguistic Inquiry and Word Count (LIWC) software (Pennebaker, Boyd, Jordan, & Blackburn, 2015). The LIWC counts how many words per input text belong to predefined psycholinguistic lexicon categories and has been used for verbal deception research before (e.g., Bond et al., 2017). For the current experiment, we used the categories “time” (e.g., “once” and “since”) and “space” (e.g., “above” and “outside”) each of which is standardized by the word count per statement and question type. We used the Dutch translation of the 2007 LIWC version (Boot, Zijlstra, & Geenen, 2017).

## 3 RESULTS

### 3.1 Preregistered analyses

#### 3.1.1 Replication

For the sake of exactly replicating the original analysis, we first tested for the Veracity main effect for the time schedule question only. There was no significant difference in specific time references between truthful and deceptive answers, *t*_*one‐sided*_(74.97) = −0.64, *p* = 0.262, *d* = −0.14 [95% CI: −0.59; 0.30]. 1Note that we standardized for the word count (as per the preregistration). In the original study, the dependent variable was not divided by the number of words, but instead the number of words was added as a covariate. Including word count as a covariate in an analysis of the uncorrected dependent variable did not change the results. There was no significant main effect of Veracity, *F*(1, 76) = 0.08, *p* = 0.785, *f* = 0.03; despite the significant effect of the covariate “word count,” *F*(1, 76) = 9.97, *p* = 0.002, *f* = 0.36. When we use the nonstandardized values for the replication analysis (*t* test), we obtain very similar results, *t*_*one‐sided*_(67.10) = −0.26, *p* = 0.396, *d* = −0.06 [95% CI: −0.50; 0.38].


The 2 (Veracity: truthful vs. deceptive, between‐subjects) by 2 (Question focus: time schedule vs. route description) mixed analysis of variance (ANOVA) on the proportion of human‐coded “specific times” showed no significant main effect of Veracity, *F*(1, 77) = 0.41, *p* = 0.525, *f* = 0.07, and no significant Veracity*Question focus interaction, *F*(1, 77) = 0.40, *p* = 0.528, *f* = 0.07. A significant main effect of Question focus, *F*(1, 77) = 23.61, *p* < 0.001, *f* = 0.55, indicated that answers to the time schedule question (*M* = 1.01, *SD* = 1.74) contained more specific times than those on the route description question (*M* = 0.04, *SD* = 0.24, see Table 2). Thus, although the time question elicited more specific time answers than the route question, we did not find that the time schedule question elicited more specific times in truth‐tellers than in liars.

**Table 2:** Means (*SD*s) per dependent variable, veracity, and question focus

Dependent variable	Time schedule question		Route description question	
	Truthful	Deceptive	Truthful	Deceptive
Human‐coded specific times	0.88 (1.67)	1.14 (1.82)	0.04 (0.23)	0.04 (0.25)
Human‐coded spatial details	4.83 (2.68)	6.70 (3.59)	10.08 (6.67)	12.60 (5.17)
LIWC‐coded temporal details	6.73 (3.93)	6.07 (4.62)	4.44 (4.48)	4.37 (3.84)
LIWC‐coded spatial details	1.80 (2.28)	2.75 (2.87)	2.96 (3.51)	3.82 (3.17)
Human‐coded temporal details	8.12 (4.35)	7.87 (4.37)	5.26 (4.23)	5.36 (3.94)
Number of words	58.32 (32.90)	58.13 (30.51)	49.90 (23.11)	42.61 (34.65)

*Note*. LIWC: Linguistic Inquiry and Word Count.

#### 3.1.2 Additional measure: Motivation

Participants were highly motivated to provide a convincing story, and the self‐reported motivation (on a scale from 0 to 10) did not differ between the two Veracity conditions (truthful: *M* = 8.37, *SD* = 1.18; deceptive: *M* = 8.17, *SD* = 0.97), *F*(1, 77) = 0.64, *p* = 0.428, *f* = 0.09. The majority of participants had made the trip before at least once (82.28%), but this did not differ between the two conditions, *X* ^2^(1) = 1, *p* = 0.999 (liars: 82.58%; truth‐tellers: 82.94%).

#### 3.1.3 Beyond replication: Additional (route) question

The 2 by 2 mixed ANOVA on the proportion of human‐coded spatial details indicated a significant main effect of Veracity, *F*(1, 77) = 8.68, *p* = 0.004, *f* = 0.36, suggesting that—contrary to the expectation—deceptive answers (*M* = 9.65, *SD* = 6.10) contained more spatial details than truthful ones (*M* = 7.46, *SD* = 4.87) regardless of Question focus. A significant main effect of Question focus, *F*(1, 77) = 52.51, *p* < 0.001, *f* = 0.82, showed that answers to the route description question (*M* = 11.29, *SD* = 6.04) contained more spatial details than those to the time schedule question (*M* = 5.73, *SD* = 3.27). The interaction was not significant, *F*(1, 77) = 0.18, *p* = 0.674, *f* = 0.05.

#### 3.1.4 Beyond replication: Additional (computerized) coding

For the LIWC‐coded temporal details, there was only a significant Question focus main effect, *F*(1, 77) = 10.24, *p* = 0.002, *f* = 0.36, showing that there were more temporal details for the time schedule question (*M* = 6.41, *SD* = 4.26) than for the route description question (*M* = 4.41, *SD* = 4.16). There was no significant Veracity main effect, *F*(1, 77) = 0.26, *p* = 0.613, *f* = 0.06, nor a significant Veracity by Question focus interaction, *F*(1, 77) = 0.22, *p* = 0.639, *f* = 0.05.

Likewise, for the LIWC‐coded spatial details, there was no Veracity main effect, *F*(1, 77) = 3.04, *p* = 0.085, *f* = 0.20, and no significant interaction effect between Veracity and Question focus, *F*(1, 77) = 0.01, *p* = 0.911, *f* = 0.01. A significant Question focus main effect, *F*(1, 77) = 6.94, *p* = 0.010, *f* = 0.30, indicated that answers to the route description question contained more spatial details, (*M* = 3.37, *SD* = 3.34) than to the time schedule question (*M* = 2.26, *SD* = 2.61).

## 4 NON‐PREREGISTERED ANALYSES

### 4.1 Bayesian hypothesis testing

#### 4.1.1 Uninformed priors

An alternative way of testing the findings is using Bayesian statistics, which is better equipped of capturing uncertainty in the data (e.g., due to small sample sizes, Lee & Wagenmakers, 2013) and is therefore able to provide more reliable estimates of, for example, mean differences between groups (Wagenmakers, Wetzels, Borsboom, & van der Maas, 2011; Wetzels et al., 2011). Moreover, Bayesian testing quantifies how likely the data are under two competing hypotheses and can, therefore, indicate evidence for the null hypothesis. Using Bayesian hypothesis testing with the *BayesFactor* R package using default, uninformed priors (Morey, Rouder, Love, & Marwick, 2015), the present study's results for the key effect indicated a Bayes factor, *BF*_01_ = 3.57 (i.e., that data were 3.57 times more likely under the null hypothesis than under the alternative hypothesis). This Bayes factor can be interpreted as substantial evidence for the null hypothesis that the truthful statements do not differ from deceptive ones over the alternative hypothesis (Wagenmakers et al., 2011). In the original study, there was substantial evidence in favor of the alternative hypothesis, *BF*_10_ = 3.24.

#### 4.1.2 Informed priors

When one possesses evidence about the likelihood of an effect before obtaining new data, this prior belief should be explicitly incorporated into the Bayesian estimation. To do so, we treat the findings of the original study as the prior evidence for the data from the replication study, which is the posterior distribution of the original study becomes the prior for the replication (Gronau, Ly, & Wagenmakers, 2017). In doing so, we incorporate the belief of the original study (i.e., that there is a moderately sized effect) into the hypothesis testing of the replication and obtain *BF*_01_ = 24555.02—“extreme evidence” in favor of the null.

Treating the original effect size (here *d* = 0.54) at face value can be misleading because most published effect sizes are overestimations of the true effect (Gelman & Carlin, 2014; Simonsohn, 2015). 2We thank Timothy Luke for pointing us in to that direction during the reviewing process. To avoid inflating evidence for the null hypothesis, we also calculated the informed prior Bayes factor estimation using a corrected original effect size of 75% (*d* = 0.41, *BF*_01_ = 4.23), 50% (*d* = 0.27, *BF*_01_ = 2.41), 25% (*d* = 0.14, *BF*_01_ = 1.59), and 10% of the original (*d* = 0.05, *BF*_01_ = 1.33). The evidence in favor of the null is inconclusive for these downward‐corrected priors. This suggests that with these corrected informed priors, our current study cannot ascertain the existence or absence of an effect that is a lot smaller than the one suggested in the original paper.

### 4.2 Further analyses

#### 4.2.1 Temporal details

We explored whether the human‐coded temporal details (i.e., including nonspecific time references such as “then” and “after”) could help discriminate truthful from deceptive statements. There was only a significant main effect of Question focus, *F*(1, 77) = 20.12, *p* < 0.001, *f* = 0.51 (time schedule: *M* = 8.00, *SD* = 4.34; route: *M* = 5.31, *SD* = 4.05).

#### 4.2.2 Statement length

The 2 by 2 mixed ANOVA on the number of words indicated only a significant Question focus main effect, *F*(1, 77) = 11.22, *p* = 0.001, *f* = 0.38. Answers to the time schedule question were lengthier (*M* = 58.23, *SD* = 31.57) than to the route description question (*M* = 46.39, *SD* = 29.71). This finding might be due to the order effects: To adhere to the procedure of the original experiment, the time schedule question always came first.

## 5 DISCUSSION

This paper presents the first replication study in the field of verbal deception detection research. The original study found that truth‐tellers mentioned more specific times than liars when talking about a trip they made. We were not able to find significant differences in the occurrence of specific times between truth‐tellers and liars.

### 5.1 Did the findings replicate?

A judgment of the success of a direct replication should go beyond mere statistical significance testing (Nosek & Errington, 2017; Open Science Collaboration, 2015). We evaluate the current replication efforts utilizing five criteria proposed by the OSC, 2015. (a) *Does the replication produce a statistically significant effect in the same direction as the original?* No. The original experiment yielded a significant difference in specific times, so that truthful statements contained more than deceptive ones (*d* = 0.54), whereas the replication effect albeit nonsignificant was in the opposite direction (*d* = −0.14). (b) *Is the effect size in the replication similar to the effect size in the original?* No. The original study showed a medium effect (Cohen's *f* = 0.27; Cohen's *d* = 0.54), whereas we obtained no significant effect (*f* = 0.07; *d* = −0.14). Bayesian analysis suggested that there was substantial (uninformed priors: *BF*_01_ = 3.58) to extreme evidence (informed *prior of original study effect size*: *BF*_01_ = 24555.02) in favor of the null hypothesis of no truth–lie difference in specific time occurrences. This is in contrast to the original study, which had *BF*_10_ = 3.24 indicating substantial evidence in the opposite direction (i.e., in favor of the alternative hypothesis). It is important to note, however, that using downward‐corrected original effect sizes for the informed priors led to inconclusive Bayes factors weakening the evidence for the null considerably. Nevertheless, these findings too would suggest that the original effect is not replicated: The true effect is either nonexistent or substantially smaller than suggested. (c) *Does the original effect size fall within the confidence or prediction interval of the replication (and vice versa)?* No. When recalculating the effect size of the original to Cohen's *d*, we obtain an effect size of *d* = 0.54 with a 95% CI of [0.10; 0.99]. Compared with the one yielded in the replication, *d* = −0.14 [−0.59; 0.30], we observe that the original one does not fall into the 95% CI of the replication effect, nor vice versa. (d) *Does a meta‐analytic combination of results from the original experiment and the replication yield a statistically significant effect?* No. Although desirably conducted with many replication studies from multiple labs replication, we ran a mini‐meta‐analysis using the original and the replication study (Valentine, Pigott, & Rothstein, 2010). The average effect size was *d* = 0.20 with a 95% CI containing zero [−0.47; 0.88]. Bayes factor estimation for the meta‐analytic result of the two *t*‐statistics indicated *BF*_01_ = 3.64—substantial evidence in favor of the null hypothesis that there is no meta‐analytical effect. (e) *Do the results of the original experiment and the replication appear to be consistent?* This question pertains to the qualitative assessment of the researcher. Each author of the present study was asked “Did the results replicate the original effect?” Out of four authors, none voted “Yes,” three voted “No,” and one voted “inconclusive.” The inconclusive vote was motivated by the low power (calculated a priori for a power of 0.80; post hoc reached power for *d* = 0.54: 0.77, see below). In addition to these five criteria, Bayesian hypothesis testing tends to favor the null hypothesis over the original hypothesis. Taken together, several assessment criteria suggest that the original study did not replicate.

### 5.2 Differences between original and replication study

We see at least *three* differences between the original and the replication that may explain the divergent findings. First, in the replication, the majority of participants reported that they had made the same trip before. This might have enabled the liars to use previous travels as a lie. In doing so, their lie contains many truthful aspects retrieved from previous experience. Although this is certainly ecologically valid, it is in stark contrast to experimental deception research where the lie is often a complete lie without resorting to previous experience (e.g., Sooniste, Granhag, Strömwall, & Vrij, 2015). The low proportion of passengers who did not make the trip before and the lack of that information from the original study do not allow us to further explore this explanation. The travellers' experience with their destination and travel to it might even be a crucial moderator (e.g., Warmelink, Vrij, Mann, Jundi, & Granhag, 2012). Clearly, more research is needed on this matter.

Second, an important aspect of direct replications is that of the setting, population, and time, so that “[e]xact replications are replications of an experiment that operationalize both the independent and the dependent variable in exactly the same way as the original study” (Stroebe & Strack, 2014, p. 61). Although the setting (on a ferry) was mirrored closely, one important difference could have been the participants' native language. In the original study, participants were interviewed in their native English language whereas the replication did so with participants in their native Dutch language. In the absence of evidence that the English and Dutch language differ in their prevalence of specific time references (for an examination of spatial references, see Van Staden, Bowerman, & Verhelst, 2006, who show that Dutch might be richer in spatial description grammar), we argue that it is unlikely that the current language differences have affected the chance of replication. Moreover, the underlying theories (e.g., Reality Monitoring) are not limited to a particular language but rather assume that the memory recollection processes are universal. 3There is evidence that Reality Monitoring and Criteria‐based Content Analysis, for example, work in Dutch participants (Bogaard, Meijer, & Vrij, 2014) and the replication study did not differ to the original in participants' language proficiency (i.e., we did not interview participants in a foreign language). Most importantly, even if the language differences between original and replication would have affected the occurrence of specific time references, this should have played an equal role for truth‐tellers and liars (see Taylor, Larner, Conchie, & Menacere, 2017).

Third, the answers given by the participants were shorter in the original (number of words *M* = 33.70, *SD* = 20.71) than in the replication (*M* = 52.31, *SD* = 31.13). Although it is not clear what caused the lengthier answers in the replication, it is possible that the differences mentioned above played a role so that, for example, participants were more talkative because they already made the trip. Importantly, however, that difference in answer length should not have lowered that chance for replication as lengthier statements are typically better suited for verbal deception detection than shorter ones (Vrij et al., 2015) and several methods are specifically designed to elicit lengthier and richer verbal accounts (e.g., the model statement technique, Harvey, Vrij, Leal, Lafferty, & Nahari, 2017).

Despite the seemingly minor (or no) detrimental effects of potential slight deviations for the original, it cannot be established whether these minor variations combined made the replication less likely. In the absence of evidence that such slight variations could have affected the findings, we acknowledge this possibility but cannot suggest which variation or which combination of variations caused the replication failure. To our best knowledge and intention, the current replication study is identical to the original in that we operationalized the independent and dependent variables precisely as was done in the original. We, therefore, deem it fair to call the replication a direct one.

### 5.3 Statistical power for the replication study

An important methodological aspect of replication efforts is the statistical power of the replication study (i.e., the likelihood that a significant effect of a given size—here: *d* = 0.54—is observed given the sample size and alpha significance threshold, Lakens, 2013). To give the original effect the best chance of replicating, the likelihood of detecting a significant effect of similar size *if it were there* should be high (=high statistical power). Statistical power depends not only on sample size and the alpha threshold but also on the effect size. Because reported effect sizes are often overestimations of a true effect (Bakker, van Dijk, & Wicherts, 2012; Simonsohn, 2015), it would be desirable to use, for example, the lower bounds of the effect size CI. In the current study, an ideal scenario with a power of 0.95, an alpha threshold of 0.05 (or smaller), and an effect size of *d* = 0.10, would require at least a sample size of 4,332 (one‐sided comparison). Simonsohn (2015) suggested that the effect size used for replication sample size calculations could best be determined by first calculating the effect size, which the original study would have detected with a power of 0.33 (*here*: d = 0.27, and required *n* = 588 for a power of 0.95).

Practical considerations in the current replication study led us to decide to mirror the identical sample size of the original study, which coincided with a priori calculations for a power of 0.80. The achieved power was marginally smaller (0.77). However, this implies that on average in the long run, the chance of observing the original effect *if it were there* was only 0.77. This implies that a single replication attempt, with a chance of 23% of incorrectly not detecting an existing effect of the original size, is not enough to conclude that the effect does not exist (at least when one would rely on the 5% significance threshold). The latter is amplified by the conclusion that most effects are overestimations, and hence, true to‐be‐replicated effects are smaller than those that are reported (Gelman & Carlin, 2014). Therefore, we can conclude that we could not replicate the original effect of identical size but we cannot with high confidence ascertain that the effect (i.e., more specific time references in truthful than in deceptive intentions) does not exist. It is possible that such an effect exists but that it is much smaller in magnitude (see also Gelman's “piranha argument” about the unlikely coexistence of large effects in behavioral science, Gelman, 2017). Taken together, if an effect is considered to be important (e.g., for practical or scientific reasons), higher powered studies and more replication attempts are needed.

### 5.4 Additional insights

We did not obtain support for the additional hypotheses that truthful statements contain more temporal details (human and computer‐coded) and more spatial details (computer‐coded) than deceptive statements. Contrary to our expectation, however, we found that deceptive statements contained more human‐coded spatial details than truthful ones. The framework of interpersonal Reality Monitoring predicts that truth‐tellers can recall an event in more detail than liars because the latter never experienced it and, therefore, have to resort to fabrication (Johnson et al., 1998; Nahari, 2018). Liars also have fewer cognitive resources available to produce a detailed, rich account of the fabricated event (Vrij & Granhag, 2012). Albeit in contradiction with this notion that liars lack the cognitive resources to produce statements as detailed as truth‐tellers, the opposite effect found for spatial details is not an exception. Previously, it has been argued that expected, factual questions are what liars prepare for and can, therefore, enrich with details (Warmelink et al., 2012). In support of that idea, people who lied about their planned weekend activities mentioned more persons and more locations than those who told the truth (Kleinberg, van der Toolen, Vrij, Arntz, & Verschuere, 2018). A working hypothesis states that liars might *overcompensate* in their statements because they are particularly inclined to appear convincing whereas truth‐tellers assume that their truth will appear naturally. In a different study, individual details mentioned by truth‐tellers and liars were coded as truthful or false and a similar pattern emerged: Liars compensated for their inability to provide sufficient truthful detail after a 2‐week delay by adding false details whereas truth‐tellers did not (Nahari, 2018). To address these dynamics, the use of unexpected questions (e.g., on the planning of the event) seems a worthwhile addition to future research on that hypothesis.

## 6 CONCLUSION

Truth‐telling and lying ferry passengers did not differ significantly in specific time references when asked about the time schedule of their travel plans. It should be noted that both the original and the replication study only provide a point estimate of the effect. This is not uncommon in replication research (e.g., Open Science Collaboration, 2015); however, ideally, any replication would consist of multiple, independent replication attempts. 4See: https://www.psychologicalscience.org/publications/replication. In the current study, the lack of high statistical power leaves the possibility that there exists an actual effect. We encourage other researchers in the deception detection community to conduct preregistered, well‐powered, multilab replication studies of the core effects of the field to consolidate the science of verbal deception detection. Such a collective effort will help clarify which effects in verbal deception research are reliable.

## References

[acp3439-bib-0001] Bakker, M. , van Dijk, A. , & Wicherts, J. M. (2012). The rules of the game called psychological science. Perspectives on Psychological Science, 7(6), 543–554. 10.1177/1745691612459060 26168111

[acp3439-bib-0002] Bogaard, G. , Meijer, E. H. , & Vrij, A. (2014). Using an example statement increases information but does not increase accuracy of CBCA, RM, and SCAN: Using an example statement with truth tellers and liars. Journal of Investigative Psychology and Offender Profiling, 11(2), 151–163. 10.1002/jip.1409

[acp3439-bib-0003] Bond, G. D. , Holman, R. D. , Eggert, J.‐A. L. , Speller, L. F. , Garcia, O. N. , Mejia, S. C. , … Rustige, R. (2017). ‘Lyin’ ‘Ted’, “Crooked Hillary,” and “Deceptive Donald”: Language of lies in the 2016 US presidential debates: Language of lies in debates. Applied Cognitive Psychology, 31(6), 668–677. 10.1002/acp.3376

[acp3439-bib-0004] Boot, P. , Zijlstra, H. , & Geenen, R. (2017). The Dutch translation of the Linguistic Inquiry and Word Count (LIWC) 2007 dictionary. Dutch Journal of Applied Linguistics, 6(1), 65–76. 10.1075/dujal.6.1.04boo

[acp3439-bib-0005] DePaulo, B. M. , Lindsay, J. J. , Malone, B. E. , Muhlenbruck, L. , Charlton, K. , & Cooper, H. (2003). Cues to deception. Psychological Bulletin, 129(1), 74–118. 10.1037/0033-2909.129.1.74 12555795

[acp3439-bib-0006] Gelman, A . (2017). The piranha problem in social psychology/behavioral economics: The “take a pill” model of science eats itself—Statistical modeling, causal inference, and social science. Retrieved June 6, 2018, from http://andrewgelman.com/2017/12/15/piranha-problem-social-psychology-behavioral-economics-button-pushing-model-science-eats/

[acp3439-bib-0007] Gelman, A. , & Carlin, J. (2014). Beyond power calculations: Assessing type S (Sign) and type M (magnitude) errors. Perspectives on Psychological Science, 9(6), 641–651. 10.1177/1745691614551642 26186114

[acp3439-bib-0008] Gelman, A. , & Loken, E. (2013). The garden of forking paths: Why multiple comparisons can be a problem, even when there is no “fishing expedition” or “p‐hacking” and the research hypothesis was posited ahead of time. Retrieved January 19, 2018, from http://www.stat.columbia.edu/~gelman/research/unpublished/p_hacking.pdf

[acp3439-bib-0009] Gronau, Q. F. , Ly, A. , & Wagenmakers, E.‐J. (2017). Informed Bayesian t‐tests. ArXiv:1704.02479 [Stat]. Retrieved from http://arxiv.org/abs/1704.02479

[acp3439-bib-0010] Harvey, A. C. , Vrij, A. , Leal, S. , Lafferty, M. , & Nahari, G. (2017). Insurance based lie detection: Enhancing the verifiability approach with a model statement component. Acta Psychologica, 174, 1–8. 10.1016/j.actpsy.2017.01.001 28088655

[acp3439-bib-0011] Johnson, M. K. , Bush, J. G. , & Mitchell, K. J. (1998). Interpersonal reality monitoring: Judging the sources of other people's memories. Social Cognition, 16(2), 199–224.

[acp3439-bib-0012] Kleinberg, B. , Mozes, M. , Arntz, A. , & Verschuere, B. (2017). Using named entities for computer‐automated verbal deception detection. Journal of Forensic Sciences 10.1111/1556-4029.13645, 63, 714–723. 28940300

[acp3439-bib-0013] Kleinberg, B. , van der Toolen, Y. , Vrij, A. , Arntz, A. , & Verschuere, B. (2018). Automated verbal credibility assessment: The model statement technique and predictive modeling. Applied Cognitive Psychology, *X*, XX–XX, 32, 354–366. 29861544 10.1002/acp.3407 PMC5969289

[acp3439-bib-0014] Lakens, D. (2013). Calculating and reporting effect sizes to facilitate cumulative science: A practical primer for t‐tests and ANOVAs. Frontiers in Psychology, 4 10.3389/fpsyg.2013.00863 PMC3840331 24324449

[acp3439-bib-0015] Lee, M. D. , & Wagenmakers, E.‐J. (2013). Bayesian cognitive modeling: A practical course. Cambridge: Cambridge University Press 10.1017/CBO9781139087759

[acp3439-bib-0016] Masip, J. , Sporer, S. L. , Garrido, E. , & Herrero, C. (2005). The detection of deception with the reality monitoring approach: A review of the empirical evidence. Psychology, Crime & Law, 11(1), 99–122. 10.1080/10683160410001726356

[acp3439-bib-0017] Morey, R. , Rouder, J. , Love, J. , & Marwick, B. (2015, September 20). Bayesfactor: 0.9.12‐2 Cran. Zenodo. 10.5281/zenodo.31202

[acp3439-bib-0018] Nahari, G. (2018). Reality monitoring in the forensic context: Digging deeper into the speech of liars. Journal of Applied Research in Memory and Cognition. 10.1016/j.jarmac.2018.04.003

[acp3439-bib-0019] Nosek, B. A. , Alter, G. , Banks, G. C. , Borsboom, D. , Bowman, S. D. , Breckler, S. J. , … Yarkoni, T. (2015). Promoting an open research culture. Science, 348(6242), 1422–1425. 10.1126/science.aab2374 26113702 PMC4550299

[acp3439-bib-0020] Nosek, B. A. , & Errington, T. M. (2017). Making sense of replications. eLife, 6 10.7554/eLife.23383 PMC5245957 28100398

[acp3439-bib-0021] Oberlader, V. A. , Naefgen, C. , Koppehele‐Goseel, J. , Quinten, L. , Banse, R. , & Schmidt, A. F. (2016). Validity of content‐based techniques to distinguish true and fabricated statements: A meta‐analysis. Law and Human Behavior, 40(4), 440–457. 27149290 10.1037/lhb0000193

[acp3439-bib-0022] Open Science Collaboration (2015). Estimating the reproducibility of psychological science. Science, 349(6251), 1–8. 10.1126/science.aac4716 26315443

[acp3439-bib-0023] Pennebaker, J. W. , Boyd, R. L. , Jordan, K. , & Blackburn, K. (2015). The development and psychometric properties of LIWC2015. Retrieved from https://repositories.lib.utexas.edu/handle/2152/31333

[acp3439-bib-0024] Simons, D. J. (2014). The value of direct replication. Perspectives on Psychological Science, 9(1), 76–80. 10.1177/1745691613514755 26173243

[acp3439-bib-0025] Simonsohn, U. (2015). Small telescopes: Detectability and the evaluation of replication results. Psychological Science, 26(5), 559–569. 10.1177/0956797614567341 25800521

[acp3439-bib-0026] Sooniste, T. , Granhag, P. A. , Strömwall, L. A. , & Vrij, A. (2015). Statements about true and false intentions: Using the Cognitive Interview to magnify the differences. Scandinavian Journal of Psychology, 56(4), 371–378. 10.1111/sjop.12216 25929812

[acp3439-bib-0027] Stroebe, W. , & Strack, F. (2014). The alleged crisis and the illusion of exact replication. Perspectives on Psychological Science, 9(1), 59–71. 10.1177/1745691613514450 26173241

[acp3439-bib-0028] Taylor, P. J. , Larner, S. , Conchie, S. M. , & Menacere, T. (2017). Culture moderates changes in linguistic self‐presentation and detail provision when deceiving others. Royal Society Open Science, 4(6), 170128 10.1098/rsos.170128 28680668 PMC5493910

[acp3439-bib-0029] Valentine, J. C. , Pigott, T. D. , & Rothstein, H. R. (2010). How many studies do you need?: A primer on statistical power for meta‐analysis. Journal of Educational and Behavioral Statistics, 35(2), 215–247. 10.3102/1076998609346961

[acp3439-bib-0030] van Elk, M. , Matzke, D. , Gronau, Q. F. , Guan, M. , Vandekerckhove, J. , & Wagenmakers, E.‐J. (2015). Meta‐analyses are no substitute for registered replications: A skeptical perspective on religious priming. Frontiers in Psychology, 6 10.3389/fpsyg.2015.01365 PMC4569810 26441741

[acp3439-bib-0031] Van Staden, M. , Bowerman, M. , & Verhelst, M. (2006). Some properties of spatial description in Dutch In Grammars of space (pp. 475–511). Cambridge, United Kingdom: Cambridge University Press.

[acp3439-bib-0032] Vrij, A. , Fisher, R. P. , & Blank, H. (2015). A cognitive approach to lie detection: A meta‐analysis. Legal and Criminological Psychology, 22(1), 1–21. 10.1111/lcrp.12088

[acp3439-bib-0033] Vrij, A. , & Granhag, P. A. (2012). Eliciting cues to deception and truth: What matters are the questions asked. Journal of Applied Research in Memory and Cognition, 1(2), 110–117. 10.1016/j.jarmac.2012.02.004

[acp3439-bib-0034] Wagenmakers, E. , Wetzels, R. , Borsboom, D. , & van der Maas, H. L. J. (2011). Why psychologists must change the way they analyze their data: The case of psi: Comment on Bem (2011). Journal of Personality and Social Psychology, 100(3), 426–432. 10.1037/a0022790 21280965

[acp3439-bib-0035] Warmelink, L. , Vrij, A. , Mann, S. , & Granhag, P. A. (2013). Spatial and temporal details in intentions: A cue to detecting deception. Applied Cognitive Psychology, 27(1), 101–106. 10.1002/acp.2878

[acp3439-bib-0036] Warmelink, L. , Vrij, A. , Mann, S. , Jundi, S. , & Granhag, P. A. (2012). The effect of question expectedness and experience on lying about intentions. Acta Psychologica, 141(2), 178–183. 10.1016/j.actpsy.2012.07.011 22964059

[acp3439-bib-0037] Wetzels, R. , Matzke, D. , Lee, M. D. , Rouder, J. N. , Iverson, G. J. , & Wagenmakers, E.‐J. (2011). Statistical evidence in experimental psychology: An empirical comparison using 855 *t* tests. Perspectives on Psychological Science, 6(3), 291–298. 10.1177/1745691611406923 26168519

